# Red Tape in Nursing Homes

**Published:** 1923-05

**Authors:** 


### Red Tape in Nursing Homes.

Sir,—It is never clear to the lay mind why it is essential for patients in a hospital to be awakened at 6 a.m. in order to undergo an unwelcome washing. Various explanations are given, but none of these convince the outsider that such torture is unavoidable. But when a patient in a nursing home is awakened at this intolerably early hour it is simply monstrous. Yet I know of a fairly big nursing home—the charges are from 5 to 8 guineas weekly—where this is the custom. Here surely no excuse can be offered, nor is it easy to see why lunch should be served at noon, tea at 3 p.m., and dinner at 6 p.m. These are not the times at which civilised persons are accustomed to have their meals. Why should hospital routine —admittedly far from ideal—be slavishly imitated by the authorities of nursing homes? Surely the patient who pays has some right to call the tune?

